# Isolated Tuberculosis of Talus: A Case Report

**DOI:** 10.5704/MOJ.1403.001

**Published:** 2014-03

**Authors:** A Dahuja, G Dahuja, R Kaur, K Bansal

**Affiliations:** Department of Orthopedics, Guru Gobind Singh Medical College, Punjab, India; Department of Orthopedics, Guru Gobind Singh Medical College, Punjab, India; Department of Orthopedics, Guru Gobind Singh Medical College, Punjab, India; Department of Orthopedics, Guru Gobind Singh Medical College, Punjab, India

## Abstract

Tuberculosis still remains a leading infection, causing death and disability worldwide. We report a patient with isolated tuberculosis of the talus bone. A 14 year old boy reported with an eight-month history of swelling and pain in his left ankle joint. Routine investigations indicated positive aetiology of tuberculous infection . Surgical curettage of the talus and, debridement were performed and a below knee POP cast was applied along with anti TB therapy. After 18 months postoperative , the patient was able to carry out his daily activities without pain .The ankle and foot are rarely affected and account for only 1% of all TB infections. Provisional diagnosis can be made through history and routine investigations but confirmation by the identification of the bacillus from the local lesion or by a histopathological examination of tissue. Talus tuberculosis should be considered in any long standing inflammatory pathology of the ankle.

**Key Words:**

Debridement, Sequester, Tuberculosis

## Introduction

Tuberculosis is still a major health problem in many developing countries. Involvement of the musculo-skeletal system is only in 1-3% of all tuberculosis patients. Most commonly, it affects the spine followed by major weight bearing joints such as the hip and knee. Isolated tuberculosis of talus is very rare with only 12 cases reported thus far in the literature^1^. Its unusual symptomatology and presentation explain its often unrecognised pathology culminating in the delay in diagnosis and treatment. We report a patient with isolated tuberculosis of the talus bone.

## CASE REPORT

**Fig. 1: X-ray of ankle joint showing lytic lesion in the talus.**
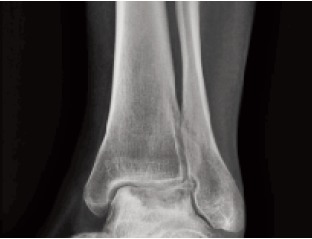


A 14 year old boy presented with an 8 month history of swelling and pain in his left ankle joint. Fever, weakness, and loss of weight were absent but there was a positive history of loss of appetite. There was no history of preceding trauma. Hemoglobin level, ESR, Mantoux test and chest X-ray were normal. On X-ray of the foot, an irregular lytic lesion of the affected part of the talus was seen (Fig. 1). MRI of the talus showed necrotic and lytic lesions over the posteromedial aspect. Ziehl-Neelsen staining of the aspirated fluid revealed acid-fast bacilli. The histological examination of the biopsy specimen showed granuloma and central caseating necrosis. Acid fast stain and PCR examination for Koch’s bacillus were positive which confirmed tuberculosis of the talus. The patient benefitted from open bone curetting and debridement through a combined anterolateral and anteromedial approach. Postoperatively, a below knee POP cast was applied for three months along with 20 months of tubercular chemotherapy which initially consisted of four drugs (Isoniazid, Rifampicin, Pyrazinamide and Ethambutol) for two months, three drugs (Isoniazid, Rifampicin,Pyrazinamide) for next six months and finally two drugs (Isoniazid, Rifampicin) for rest of twelve months. Physiotherapy was started three months post-surgery and patient was followed up monthly for the first six months and then after every three months till the completion of the chemotherapy course. Partial weight bearing was allowed after six months postoperatively and full weight bearing after nine months postoperatively. At the end of the anti-TB therapy, the patient had no pain while walking and was able to perform daily activities without restrictions.

## Discussion

Tuberculosis still remains a major infection, causing death and disability worldwide^2^. Extra pulmonary involvement is noted in 23-30% of patients infected with TB, with only 1- 3% having bone and joint disease. Thirty to fifty percent of patients with bone TB have vertebral involvement ^2^. Less frequently the appendicular skeleton, and usually major weight-bearing joints of the lower extremity such as hip and knee, are affected. The ankle and foot are rarely affected and account for only 1% of all TB infections^2, 3^ . In a report of 74 patients with foot or ankle TB, only one case of talus TB was reported by Dhillon et al ^2^. Symptomatology is frequently led by an insidious onset of pain in the ankle with functional disability^4^. Vague characteristics of the symptoms explain the difficulty and delay in diagnosis, also observed by Anderson^3^. X-rays may also be nonspecific. It can be normal at the early stage, as in our case. Subsequently signs of bone destruction and osteolysis appear ^5^. The CT scan and Magnetic Resonance Imaging (MRI) have roles in making the early diagnosis in such unusual sites. CT scan reveals the extension of lesions and bony destruction. MRI shows bone destruction sites at a precocious stage^5^. Similar MRI findings can also be seen in osteochondritis dissecans of the talus. So confirmation is only by identifying the bacillus from the local lesion or by a histopathological study of the sequestra^4^ . The aim of surgical treatment is two-fold. Firstly, the diagnosis is arrived at through obtaining tissue for bacteriological and histological study and secondly treatment is also supplemented through curettage of the diseased part in the bone. This treatment should always be complemented with plaster cast immobilization for a period of three months, followed by physiotherapy^4^. The treatment was completed with 18-20 months of anti-TB drug regime with favorable outcome despite the delay in diagnosis.

Chemotherapy was instituted for a longer period primarily in consideration of the increased prevalence of tuberculosis in India. The prognosis in this disease and its resolution depends on early diagnosis and treatment. Talus tuberculosis and the above mentioned treatment for tuberculous osteitis of the talus should be considered in any long-standing inflammatory symptoms in the ankle. The symptoms are often vague, leading to late diagnosis but favourable outcome can be achieved with surgical treatment and prompt chemotherapy.

**Fig. 2: Showing posteromedial necrosis on MRI of the talus.**
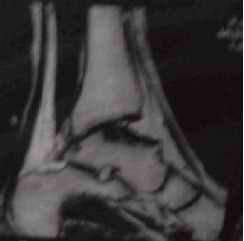


**Fig. 3: Histopathology of the talus showing caseating granuloma.**
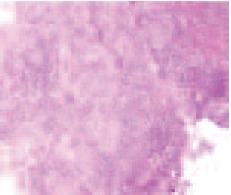


## Conclusion

Conservative management of displaced two-part fractures of the humeral neck in elderly patients is a safe, efficacious, and acceptable mode of treatment. On final follow-up at 12 months post-injury, 42 out of 48 patients (88%) were satisfied with their outcome and reported that they would choose to undergo the same treatment if they had to do everything all over again. More comparative studies between conservative and operative management may be needed before justifying the added morbidity and expense associated with surgical intervention. Further studies could also address the limitations encountered in this study, especially the lack of ancillary procedures that could have helped point out the reasons for some patients having poorer outcome than others at final follow-up.
